# Sodium–Calcium Exchanger Can Account for Regenerative Ca^2+^ Entry in Thin Astrocyte Processes

**DOI:** 10.3389/fncel.2018.00250

**Published:** 2018-08-14

**Authors:** Alexey R. Brazhe, Andrey Y. Verisokin, Darya V. Verveyko, Dmitry E. Postnov

**Affiliations:** ^1^Department of Biophysics, Biological Faculty, Lomonosov Moscow State University, Moscow, Russia; ^2^Department of Theoretical Physics, Kursk State University, Kursk, Russia; ^3^Department of physics, Saratov State University, Saratov, Russia

**Keywords:** astrocytic calcium, sodium-calcium exchanger, NCX, calcium-induced calcium entry, calcium wave

## Abstract

Calcium transients in thin astrocytic processes can be important in synaptic plasticity, but their mechanism is not completely understood. Clearance of synaptic glutamate leads to increase in astrocytic sodium. This can electrochemically favor the reverse mode of the Na/Ca-exchanger (NCX) and allow calcium into the cell, accounting for activity-dependent calcium transients in perisynaptic astrocytic processes. However, cytosolic sodium and calcium are also allosteric regulators of the NCX, thus adding kinetic constraints on the NCX-mediated fluxes and providing for complexity of the system dynamics. Our modeling indicates that the calcium-dependent activation and also calcium-dependent escape from the sodium-mediated inactive state of the NCX in astrocytes can form a positive feedback loop and lead to regenerative calcium influx. This can result in sodium-dependent amplification of calcium transients from nearby locations or other membrane mechanisms. Prolonged conditions of elevated sodium, for example in ischemia, can also lead to bistability in cytosolic calcium levels, where a delayed transition to the high-calcium state can be triggered by a short calcium transient. These theoretical predictions call for a dedicated experimental estimation of the kinetic parameters of the astrocytic Na/Ca-exchanger.

## 1. Introduction

Astrocytes are tightly involved in the metabolic supply in the brain, life cycle of the synapse and neuronal activity (Prebil et al., 2011; Lee et al., 2014; Verkhratsky and Nedergaard, 2014; Rose and Chatton, 2016). Calcium transients, engulfing areas from single astrocyte processes to whole spatial domains occupied by a single astrocyte to intercellular waves of elevated Ca^2+^, play a central role in shaping astrocytic signals to the neural tissue (Khakh and McCarthy, 2015; Bazargani and Attwell, 2016; Verkhratsky et al., 2017). If Ca^2+^ transients are the keystone of the astrocyte-mediated regulatory pathways, it is crucial to understand the mechanisms of transient origination and expansion to neighboring regions, as well as interplay between multiple mechanisms. Because thin astrocytic processes (leaflets in terminology suggested by Tong et al., 2013; Khakh and Sofroniew, 2015) are the main “sensors” of the neuronal microenvironment and the most active Ca^2+^ signaling occurs there, the mechanisms underlying formation of this activity are of key importance, but are still not clear. So, how does astrocyte read out ambient synaptic activity and transform it into local Ca^2+^ transients? We argue that Na/Ca-exchanger (NCX)—a membrane transporter protein that exchanges three Na^+^ ions for one Ca^2+^ ion—and its modulation by cytosolic Na^+^ and Ca^2+^ have important and previously unanticipated effects on Ca^2+^ transient formation and spreading.

The most widely known mechanism for astrocytic Ca^2+^ signaling includes Ca^2+^-dependent activation of inositoltrisphosphate (IP_3_) receptors (IP_3_Rs) followed by Ca^2+^–dependent Ca^2+^ release from endoplasmic reticulum (ER). Initial IP_3_ production is attributed to activation of astrocytic G protein-coupled receptors to glutamate (mGluRs), with subsequent production of diacylglycerol and IP_3_ by phospholipase Cβ. The importance of this pathway has been challenged by questioning the existence of mGluRs in adult astrocytes (Sun et al., 2013) and the lack of effects of knocking out astrocytic IP_3_Rs on pyramidal neuron synaptic activity in hippocampus (Petravicz et al., 2008). On the other hand, contrary to somata and thick branches, spontaneous Ca^2+^ transients in the leaflets are independent of intracellular stores and IP_3_-mediated mechanisms. Transients in the leaflets remain even in IP_3_R–knockout animals (Srinivasan et al., 2015), and are not affected by a variety of blockers, while being sensitive to external Ca^2+^ concentration (Rungta et al., 2016), thus implying that Ca^2+^ must enter the leaflet cytoplasm from extracellular space. This is also supported by spatial constraints: the leaflets are so small they do not contain organelles (Patrushev et al., 2013). The dominating mechanism of Ca^2+^ transients in leaflets is debated, with tentative pathways encompassing several types of transient receptor potential (TRP) channels, astrocytic ionotropic receptors to glutamate, voltage-gated Ca^2+^ channels and NCX working in Ca^2+^ entry mode (Verkhratsky et al., 2017).

Here we focus on Ca^2+^ entry, mediated by NCX. In most conditions, the NCX utilizes the Na^+^ electrochemical gradient to expell Ca^2+^ from the cell (“forward mode”). The opposite exchange direction (“reverse mode”) becomes energetically favorable at high enough [Na^+^]_*i*_ and depolarized membrane potential. For example, in the cardiomyocytes, the NCX switches between the modes during the contraction cycle (Shattock et al., 2015). NCX-mediated Ca^2+^ entry can be directly linked to local synaptic activity with the following causal chain of events (Rojas et al., 2007; Kirischuk et al., 2012; Reyes et al., 2012): neurotransmitter clearance is mediated by cotransporter proteins utilizing Na^+^ gradient, which leads to Na^+^ influx and synaptic activity-related Na^+^ transients in astrocytes (Langer and Rose, 2009; Langer et al., 2012). In the cortex, glutamate is the primary neurotransmitter, which is cotransporter with 3 Na^+^ ions per glutamate molecule. Increase in [Na^+^]_*i*_ in turn reverses the NCX cycle direction leading to Ca^2+^ entry from the extracellular space in exchange for 3 Na^+^ ions, placing NCX as a major contributor to overall Ca^2+^ and Na^+^ homeostasis in astrocytes (Reyes et al., 2012).

This Na^+^-paved link from synaptic activity to cytosolic Ca^2+^ can partly explain why the membrane of astrocyte perisynaptic processes is enriched in NCX (Minelli et al., 2007). There is however an additional facet to this picture so far not discussed in the context of astrocytes. Cardiac NCX1 isoform is allosterically regulated by Na^+^ and Ca^2+^ from the cytoplasmic side (Hilgemann et al., 1992a,b; Matsuoka et al., 1996). Specifically, already at resting [Na^+^]_*i*_, the NCX is inhibited by Na^+^, but this Na^+^-block is relieved by intracellular Ca^2+^ at concentrations close to 1 μM. This can have important dynamical consequences for Ca^2+^ dynamics in leaflets, which we demonstrate in a simple model of NCX in terms of the Hodgkin–Huxley formalism. We conjecture that high [Na^+^]_*i*_ and depolarization conditions allow Ca^2+^-induced Ca^2+^ entry through the NCX, making it a Ca^2+^-sensitive source or even mediate a Ca^2+^ wave expansion. Our results extend the current understanding of the role of NCX in Ca^2+^ dynamics as formulated by Kirischuk et al. (2012) that “NCX provides for rapid and short-lived Ca^2+^ microdomains” by a novel regenerative Ca^2+^ entry mechanism.

## 2. Simple model of the NCX

**Figure 1:**
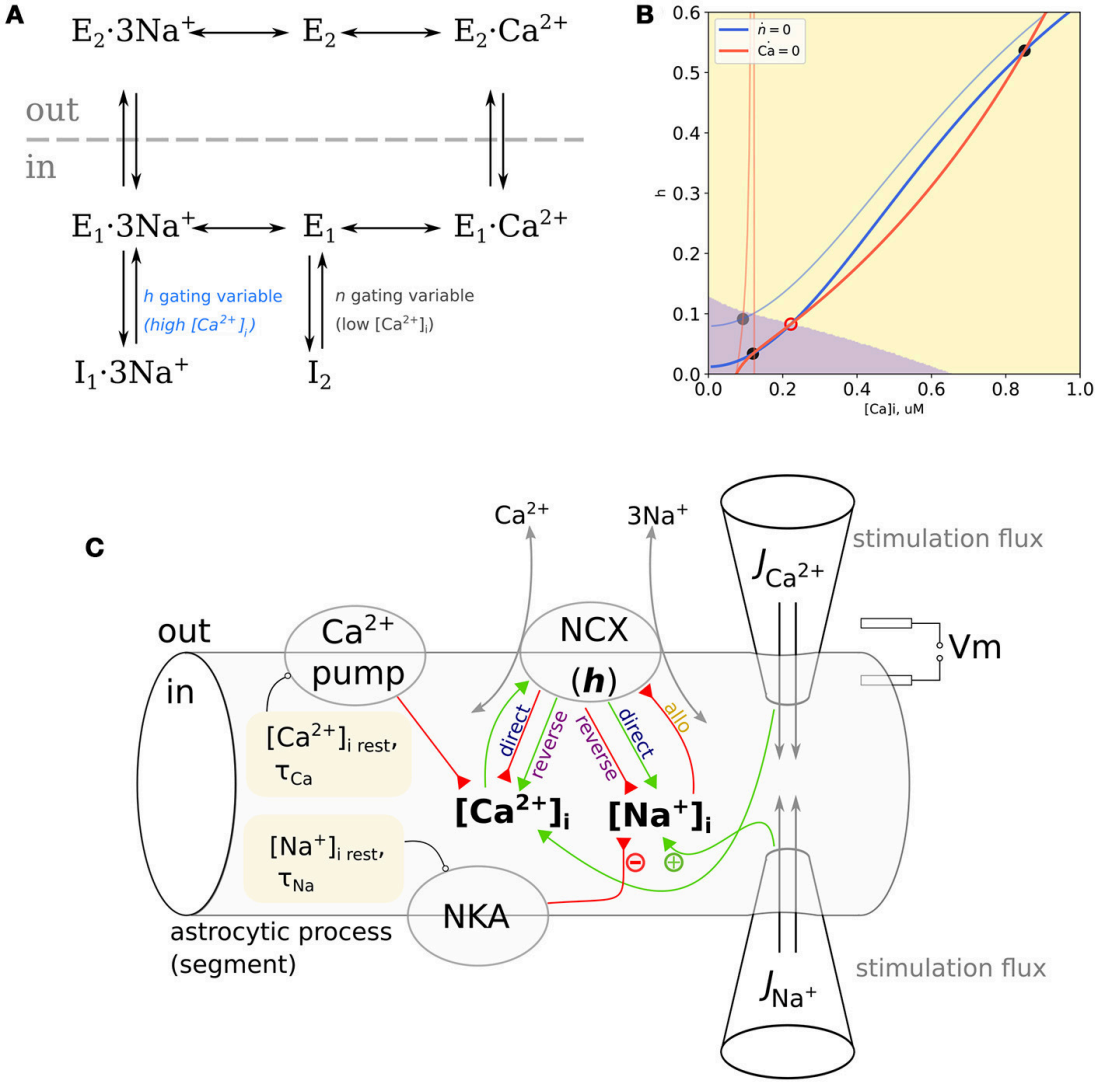
**(A)** Kinetic cycle of the NCX based on Matsuoka et al. (1996). The I_2_ inactivated state is Na^+^-independent and relieved by ${\left[\text{C}{\text{a}}^{2+}\right]}_{i}$ at ≈ 100 nM range with a fast kinetics, the I_1_ is Na^+^-dependent and relieved by ${\left[\text{C}{\text{a}}^{2+}\right]}_{i}$ at ≈ 1 μM level with a slower kinetics. **(B)** NCX as a trigger. Nullclines of the proposed model with clamped ${\left[\text{N}{\text{a}}^{+}\right]}_{i}$, blue: *dh*/*dt* = 0, red: $d{\left[\text{C}{\text{a}}^{2+}\right]}_{i}/dt=0$; pale curves: ${\left[\text{N}{\text{a}}^{+}\right]}_{i}=17$ mM, bold curves: ${\left[\text{N}{\text{a}}^{+}\right]}_{i}=45$ mM. Attraction domains corresponding to low-Ca^2+^ and high-Ca^2+^ states at ${\left[\text{N}{\text{a}}^{+}\right]}_{i}=45$ mM are shown in purple and yellow, respectively. Stable equilibria are marked with gray filled circles, an unstable equilibrium is marked with a red open circle. **(C)** Scheme of the model space, representing a small section of the thin astrocytic process. Resting values for [Na^+^] and [Ca^2+^] are set by Na^+^/K^+^-pump (NKA) and Ca^2+^-pump, NCX can exert either positive or negative influence on ${\left[\text{N}{\text{a}}^{+}\right]}_{i}$ and ${\left[\text{C}{\text{a}}^{2+}\right]}_{i}$, depending on the mode. Probing external pulses of Ca^2+^ and Na^+^ are administered through virtual “pipettes” Positive and negative effects are indicated with green and red arrows.

NCX is a reversible transporter exchanging three Na^+^ ions for one Ca^2+^ ion. Its cycle is described by a kinetic scheme shown in Figure 1A (Matsuoka et al., 1996) (counter-clockwise, “forward mode”): a substrate-free protein with outward-facing binding cite (E_2_) binds Na^+^ ions (E_2_·3Na^+^) and changes its conformation to the inward-facing binding site (E_1_·3Na^+^), from where it can either release Na^+^ to acquire the substrate-free inward-facing conformation (E_1_) or undergo transition to Na^+^-bound inactive conformation I_1_ in a Na^+^-dependent manner; the protein in E_1_ state can bind a Ca^2+^ ion (E_1_·Ca^2+^) or switch to substrate-free inactive state I_2_; the inward-facing Ca^2+^-bound state (E_1_·Ca^2+^) flips to the outward-facing Ca^2+^-bound state (E_2_·Ca^2+^), and dissociation of Ca^2+^ closes the cycle. All the transitions are reversible and going rough the cycle in the clockwise direction corresponds to “reverse” (Ca^2+^-influx) mode. Transitions from I_1_ and I_2_ are facilitated by cytosolic Ca^2+^. Thus, NCX activity is allosterically regulated by cytosolic Na^+^ and Ca^2+^ (Hilgemann et al., 1992a,b); Ca^2+^ can bind to two regulatory sites with different affinities (Boyman et al., 2009): sub-micromolar Ca^2+^ binding to the high-affinity site rapidly facilitates transition from inactive state I_2_; Na^+^-dependent transition to I_1_ is slower and the deinactivation is mediated by Ca^2+^ binding to the low-affinity Ca^2+^ site (Matsuoka et al., 1996).

We follow the general approach of earlier works by Weber et al. (2001) and Ginsburg et al. (2013) and model the NCX-mediated Ca^2+^ flow (1) as a product of maximal flow ${\bar{J}}_{\text{ncx}}$, electrochemical component *J*_Δ*E*_ (2)–(3), and gating variables *n* and *h* corresponding to deinactivation dependent on occupancy of the high- and low-affinity Ca^2+^ binding sites. The *J*_Δ*E*_ factor is described as a simplification of the normalized net reaction rate of the ping-pong bi-bi cyclic reaction scheme. In essence, *J*_Δ*E*_ models the main kinetic cycle shown in Figure 1A, while *h* and *n* correspond to the fraction of active enzyme in the exchange with *I*_1_ and *I*_2_ states, respectively.

(1) $$\begin{array}{rc} J_{\text{ncx}} & ={\bar{J}}_{\text{ncx}}n_{\infty }\left({\left[\text{Ca}\right]}_{\text{i}}\right)hJ_{\Delta E}, \end{array}$$

(2) $$\begin{array}{rc} J_{\Delta E} & =\frac{{\left[\text{N}{\text{a}}^{+}\right]}_{\text{i}}^{3}{\left[\text{C}{\text{a}}^{2+}\right]}_{\text{o}}\exp\frac{\eta VF}{RT}-{\left[\text{N}{\text{a}}^{+}\right]}_{\text{o}}^{3}{\left[\text{C}{\text{a}}^{2+}\right]}_{\text{i}}\exp\frac{\left(\eta -1\right)VF}{RT}}{K_{X}\left(1+k_{sat}\exp\frac{\left(\eta -1\right)VF}{RT}\right)}, \end{array}$$

(3) $$\begin{array}{l} K_{X}=K_{M{\text{Ca}}_{o}}{\left[\text{N}{\text{a}}^{+}\right]}_{\text{i}}^{3}+{K_{M{\text{Na}}_{o}}}^{3}{\left[\text{C}{\text{a}}^{2+}\right]}_{\text{i}}\\ \;+{K_{M{\text{Na}}_{i}}}^{3}{\left[\text{C}{\text{a}}^{2+}\right]}_{\text{o}}\left(1+\frac{{\left[\text{C}{\text{a}}^{2+}\right]}_{\text{i}}}{K_{M\text{C}{\text{a}}_{i}}}\right)\\ \;+K_{M\text{C}{\text{a}}_{i}}{\left[\text{Na}\right]}_{o}^{3}\left(1+\frac{{\left[\text{Na}\right]}_{i}^{3}}{{K_{M\text{N}{\text{a}}_{i}}}^{3}}\right)+{\left[\text{Na}\right]}_{i}^{3}{\left[\text{C}{\text{a}}^{2+}\right]}_{\text{o}}\\ \;+{\left[\text{Na}\right]}_{o}^{3}{\left[\text{C}{\text{a}}^{2+}\right]}_{\text{i}}. \end{array}$$

Here *K*_*M*·_ are the corresponding Michaelis constants, η ∈ 0 … 1 is the position of the energy barrier of the enzyme in the membrane electric field relative to membrane width, and *k*_*sat*_ parameter controls the saturation of *J*_ncx_ at negative potentials; these parameters are as in Weber et al. (2001) without change.

The allosteric factor of Weber et al. (2001) and Ginsburg et al. (2013) described binding to the high-affinity Ca^2+^-sensing site and deinactivation from I_2_. The authors assumed that [Na^+^]_*i*_ in cardiomyocytes didn't reach levels required for the transition to I_1_, which was thus not considered in their model. In contrast, Matsuoka et al. (1996) reported that Na^+^-dependent inactivation saturates at 25–50 mM [Na^+^]_*i*_, which is near the physiological range of [Na^+^]_*i*_ in astrocytes—15–20 mM (Verkhratsky et al., 2017), suggesting it to have a significant impact on astrocytic NCX activity. In a detailed experiment-based model developed by Fujioka et al. (2000) the kinetic rates of the *E*_1_ ⇌ *I*_2_ reaction are an order of magnitude higher than of the $E_{1}\cdot 3\text{N}{\text{a}}^{+}⇌I_{1}\cdot 3\text{N}{\text{a}}^{+}$ tranistion. This is also supported by Boyman et al. (2009), where the kinetics of the high-affinity Ca^2+^ binding site is also estimated to be much faster than of the low-affinity site. Correspondingly, as a simplification, we regard the *n* variable as instantaneous with the Ca^2+^-dependent steady state

(4) $$\begin{array}{r} n_{\infty }=\frac{1}{1+{\left(\frac{K_{n}}{{\left[\text{Ca}\right]}_{\text{i}}}\right)}^{2}}, \end{array}$$

and only describe kinetics of the *h* gating variable corresponding to Na^+^-dependent inactivation and Ca^2+^-dependent deinactivation mediated by the low-affinity site (5)–(7):

(5) $$\begin{array}{rc} \frac{dh}{dt} & =\left(h_{\infty }-h\right)/\tau_{h} \end{array}$$

(6) $$\begin{array}{rc} h_{\infty } & =1-\frac{1}{1+{\left({\left[\text{Ca}\right]}_{\text{i}}/K_{\text{Ca}}\right)}^{H_{Ca}}}\frac{1}{1+{\left(K_{\text{Na}}/{\left[\text{Na}\right]}_{\text{i}}\right)}^{H_{Na}}}, \end{array}$$

(7) $$\begin{array}{rc} \tau_{h} & =0.25+\tau_{0}/\left(1+{\left({\left[\text{Ca}\right]}_{\text{i}}/K_{\tau }\right)}^{H_{\tau }}\right). \end{array}$$

The steady-state value of *h* negatively depends on [Na^+^]_*i*_, but the extent of this influence is scaled by [Ca^2+^]_*i*_ with cooperativity coefficients *H*_*Na*_ and *H*_*Ca*_, correspondingly (6). We also acknowledge the dependence of τ_*h*_ on [Ca^2+^]_*i*_ reported in Matsuoka et al. (1996). This dependence is parameterized by the time scale at low [Ca^2+^]_*i*_, τ_0_, Hill coefficient *H*_τ_ and time scale at high [Ca^2+^]_*i*_, τ_*min*_ = 0.25. Qualitative reproduction of tue key experimental kinetics from Matsuoka et al. by our model is shown in Supplementary Figure 1.

Below we demonstrate that the modulation of the NCX by Na^+^ and Ca^2+^ can account for nonlinear Ca^2+^-induced Ca^2+^-entry and in bistability between low- and high-Ca^2+^ states during the high Na^+^-conditions.

## 3. Non-linear amplification of calcium transients by the NCX

### 3.1. Phaseplane analysis

We start with phaseplane analysis of a two-variable model where NCX is the only nonlinear mechanism responsible for Ca^2+^ dynamics and [Na^+^]_*i*_ is a parameter:

(8) $$\begin{array}{r} \frac{d{\left[\text{C}{\text{a}}^{2+}\right]}_{i}}{dt}=J_{\text{ncx}}+\frac{{\left[\text{C}{\text{a}}^{2+}\right]}_{\text{rest}}-{\left[\text{C}{\text{a}}^{2+}\right]}_{i}}{\tau_{\text{Ca}}}, \end{array}$$

where *J*_ncx_ is defined in (1)–(3), and for simplicity, all Ca^2+^ equilibrating mechanisms, including Ca^2+^ buffering in the excessive buffer approximation, are lumped into a single linear term setting resting Ca^2+^ concentration and equilibration timescale τ_Ca_. The second equation of the model is the kinetics of the *h* gating variable (5).

Phase plane $\left\{{\left[\text{C}{\text{a}}^{2+}\right]}_{\text{i}},h\right\}$ of this model is delineated by two nullclines, i.e., curves on the plane defined by the conditions *dh*/*dt* = 0 (*h*-nullcline), and $d{\left[\text{C}{\text{a}}^{2+}\right]}_{\text{i}}/dt=0$ (Ca^2+^-nullcline). Any point on this plane corresponds to some state of the model, a combination of specific [Ca^2+^]_*i*_ and *h* values. Dynamics of the model starting from some initial conditions will be a line on this plane, called phase trajectory. Because $d{\left[\text{C}{\text{a}}^{2+}\right]}_{\text{i}}/dt=0$ in all points lying on the Ca^2+^-nullcline, all trajectories will cross it vertically. Correspondingly, all trajectories must cross the *h*-nullcline horizontally. Nullcline intersections are equilibrium points because here both variables don't change: $d{\left[\text{C}{\text{a}}^{2+}\right]}_{\text{i}}/dt=dh/dt=0$. Equilibria can be stable if small perturbations from it relax back to the equilibrium or unstable if a small perturbation grows in time. When there are several stable equilibria on the plane, the system is multistable, and it's state space is delineated by basins of attraction of the stable equilibria. Change in parameters can shift and bend nullclines leading to changes in the system behavior.

While *h*-nullcline (defined by *h* = *h*_∞_) shows only subtle dependence on [Na^+^]i, the Ca^2+^-nullcline is more parameter-sensitive. First, it has a discontinuity when *J*_Δ*E*_=0 which implies

(9) $$\begin{array}{rc} \frac{{\left[Na^{+}\right]}_{i}^{3}}{{\left[Na^{+}\right]}_{o}^{3}} & =\frac{{\left[\text{C}{\text{a}}^{2+}\right]}_{\text{i}}}{{\left[\text{C}{\text{a}}^{2+}\right]}_{\text{o}}}\exp-\frac{VF}{RT}. \end{array}$$

Because when *J*_ncx_ vanishes, the $d{\left[\text{C}{\text{a}}^{2+}\right]}_{\text{i}}/dt$ generally does not, this discontinuity does not affect the dynamics under study. Second, the curvature near the resting Ca^2+^ range and slope of the Ca^2+^-nullcline considerably depend on the ${\bar{J}}_{\text{ncx}}$, [Na^+^]_*i*_, and *V*_*m*_.

Consequently, at low [Na^+^]_*i*_ for any reasonable ${\bar{J}}_{\text{ncx}}$, as well as low ${\bar{J}}_{\text{ncx}}$ and variable [Na^+^]_*i*_, there is a single and stable equilibrium point. However, the appropriate combination of high [Na^+^]_*i*_, depolarized *V*_*m*_ and ${\bar{J}}_{\text{ncx}}$ result in three intersection points, thus indicating bistability and a trigger-like behavior. Both cases are illustrated in Figure 1B at resting [Na^+^]_*i*_ (20 mM, pale lines) the Ca^2+^–nullcline shows a discontinuity within a physiological range of resting [Ca^2+^]_*i*_ and a single equilibrium point. Increasing [Na^+^]i to 80 mM tilts the Ca^2+^–nullcline, and drives the discontinuity to very high values of [Ca^2+^]_*i*_. It leads to appearance of two stable equilibrium points: one at low [Ca^2+^]_*i*_ and one at higher [Ca^2+^]_*i*_. All trajectories starting from the purple region of the phase plane will converge to the low–Ca^2+^ state, while all trajectories starting from the yellow region will converge to the high–Ca^2+^ state.

Because both *V*_*m*_ and [Na^+^]_*i*_ shape the Ca^2+^-nullcline, a smaller increase in [Na^+^]_*i*_ would lead to a similar nullcline configuration at a more depolarized *V*_*m*_. Maps of the highest stable [Ca^2+^]_*i*_ and regions of *V*_*m*_–[Na^+^]_*i*_ combinations leading to bistability for different ${\bar{J}}_{\text{ncx}}$ values are shown in Supplementary Figure 2. In summary, phase plane analysis predicts that the Na^+^ and Ca^2+^ modulation of the NCX leads to bistability in the system with NCX shaping the Ca^2+^ exchange.

### 3.2. Sodium-dependent amplification of calcium transients by NCX

We next turn to a simple point model with both [Na^+^]_*i*_ and [Ca^2+^]_*i*_ as dynamical variables and an additional controlled Ca^2+^-flux $J_{\text{ext}}^{\text{Ca}}$ thus extending (8) to look like:

(10) $$\begin{array}{r} \frac{d{\left[\text{C}{\text{a}}^{2+}\right]}_{i}}{dt}=J_{\text{ncx}}+J_{\text{ext}}^{\text{Ca}}+\frac{{\left[\text{C}{\text{a}}^{2+}\right]}_{\text{rest}}-{\left[\text{C}{\text{a}}^{2+}\right]}_{i}}{\tau_{\text{Ca}}} \end{array}$$

and add a simple dynamics for Na^+^ also with a controlled Na influx $J_{ext}^{\text{Na}}$:

(11) $$\begin{array}{r} \frac{d{\left[\text{N}{\text{a}}^{+}\right]}_{i}}{dt}=J_{\text{ext}}^{\text{Na}}-3J_{\text{ncx}}+\frac{{\left[\text{N}{\text{a}}^{+}\right]}_{\text{rest}}-{\left[\text{N}{\text{a}}^{+}\right]}_{i}}{\tau_{\text{Na}}}. \end{array}$$

**Figure 2:**
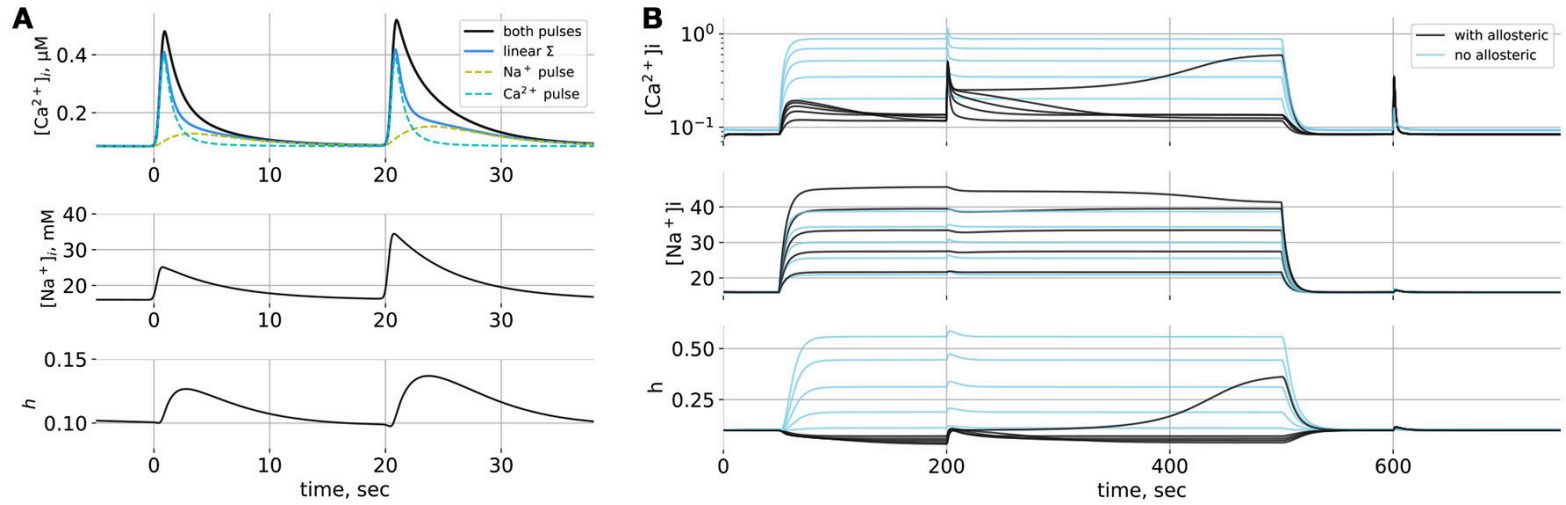
**(A)** Simulations of the point model. **(A)** Responses to short Na^+^-pulses. Solid black line: reponse to simultaneous Ca^2+^ and Na^+^ pulses, dashed lines: responses to separate Ca^2+^ and Na^+^ pulses, solid blue line: linear summation of independent responses. The two Na^+^ pulses differ in amplitude. **(B)** Responses to prolonged Na^+^ elevations of different amplitudes; Na^+^ influx lasts from *t* = 50 to *t* = 500 s; at *t* = 200 s and *t* = 600 s short Ca^2+^ pulses are given. Black lines: NCX model with allosteric regulation by Na^+^ and Ca^2+^, blue lines: NCX model without allosteric regulation.

Model space is summarized in Figure 1C and simulation of this model is shown in Figure 2. When only short Na^+^ pulses are given (Figure 2A), the system responds with small elevations of Ca^2+^ (yellow dashed line). When short Ca^2+^ pulses are given simultaneously with Na^+^ pulses, the resulting Ca^2+^ transients are amplified nonlinearly, more than can be explained by a simple summation of responses to separate Ca^2+^ and Na^+^ pulses (blue line). A smaller Na^+^ pulse (first) leads to a less pronounced effect than a larger one (second), the difference being primarily in the duration of the resulting Ca^2+^ transient. Short Na^+^ pulses cannot demonstrate bistability of the model, because most trajectories will have to pass near the nullclines (Figure 1B) resulting in slow system dynamics. The extent to which NCX can widen Ca^2+^ transients can be demonstrated with long Na^+^ pulses (Figure 2B). Here 200–s Na^+^ pulses lead to prolonged Na^+^ elevations to different levels, and the model responses (black lines) are compared to a model where allosteric modulation is turned off and ${\bar{J}}_{\text{ncx}}$ is reduced to compensate for higher ratio of active transporters (blue lines). The situation of long-term Na^+^ elevation can also reflect a pathological condition, for example prolonged ischemia, when the Na/K–pump activity is challenged by shortage in ATP or epileptic activity of neurons, leading to a sustained influx of Na^+^ into astrocytes with glutamate. Astrocytic [Ca^2+^]_*i*_ responds to the onset of $J_{\text{ext}}^{\text{Na}}$ with a small transient which returns to resting [Ca^2+^]_*i*_ due to Na^+^-dependent drop in *h*. A short Ca^2+^ stimulating pulse is widened by NCX in the Na^+^-dependent manner due to a regenerative Ca^+^ influx, where rising [Ca^2+^]_*i*_ deinactivates NCX (increases *h*) with a positive feedback. Eventually, a high enough increase in [Na^+^]_*i*_ is sufficient to lead to a sustained high–Ca^2+^ state. Note,that activation of NCX by a Ca^2+^ pulse also leads to a drop in [Na^+^]_*i*_ level. The same Ca^2+^ pulse given without Na^+^ leads only to a small Na^+^ transient due to NCX working in the direct mode.

Will the same mechanism allow for spreading waves of Ca^2+^ elevation in a spatially distributed system? We tested this conjecture in a minimalistic one-dimensional model describing a section of a thin astrocytic process lacking internal Ca^2+^ stores. One-dimensional space is discretized into elements 0.35 μm long. Each element contains the same local dynamics as in the point model with an additinal diffusion of Na^+^ and Ca^2+^ between the elements. Only the central (*x* = 0) element experienced an external Ca^2+^-pulse and elements from *x* = 0 to *x* = 10μm. on the one side experienced a short Na^+^ pulse. Ca^2+^ could diffuse in both directions from the central position, but [Na^+^]_*i*_ profile was uneven, leading to anisotropic amplification and spreading of the initial Ca^2+^ pulse as compared to linear summation of the [Ca^2+^]_*i*_ changes caused by Ca^2+^ and Na^+^ pulses given separately (Supplementary Figure 3).

## 4. Discussion

We argue that Na^+^ influx caused by neurotransmitter uptake, if happening under resting [Ca^2+^]_*i*_-conditions, beside a small elevation in [Ca^2+^]_*i*_, can “prime” the NCX by simultaneously electrochemically favoring Ca^2+^ influx via the reverse mode and inactivating NCX by Na^+^-dependent transition to *I*_1_. In this state, a localized Ca^2+^ surge, e.g., via spontaneous opening of a TRP channel, will be amplified nonlinearly by the NCX rescued form the inactive conformation by Ca^2+^. In contrast, under resting membrane potential and low [Na^+^]i, localized Ca^2+^ transients will be reduced by NCX working in the direct mode. This attenuation can be small if a large portion of the NCX is already inactivated at resting [Na^+^]i, but can be substantial in sub-normal [Na^+^]_*i*_ conditions.

Our modeling predicts that NCX can increase amplitude and duration of Ca^2+^ transients, account for switching between low-Ca^2+^ and high-Ca^2+^ states and even spatially spreading zones of elevated Ca^2+^. The extent and duration of Na^+^ elevations are dependent on the timescale of Na^+^ clearance from the cytoplasm by the Na/K-pump. In hippocampal slices (Langer et al., 2012, 2016) activity-dependent Na^+^ elevations were observed on a similar timescale to the presented in simulations. On this timescale the NCX-mediated Ca^2+^ influx can prime IP_3_ receptors on ER membranes in the astrocytic branchlets and branches in a fashion, similar to that proposed in cardiomyocytes, where the reverse mode of NCX is supposed to participate in excitation–contraction coupling (Shattock et al., 2015). So, NCX can serve as a nonlinear link from synaptic activity to the complex Ca^2+^ dynamics of the astrocyte. This, especially in view of apparent colocalization between NCX and IP_3_-receptors in plasma membrane-ER junctions (Blaustein et al., 2002), calls for further theoretical investigation of interaction between the store-operated Ca^2+^ dynamics and the NCX-mediated Ca^2+^ influx. It is also expected that under a metabolically challenged state, when the Na/K-pump turnover is slowered, there will be higher Na^+^ elevations and correspondigly higher NCX-mediated Ca^2+^ influx. Corroborating this prediction, a recent study by Gerkau et al. (2017) demonstrated pronounced Na^+^ loading and increased Ca^2+^ transients in astrocytes and neurons under chemical ischemia; blocking the reverse mode of NCX increased Na^+^ loading and dramatically reduced Ca^2+^ activity. Pathologically high synaptic activity is also expected to both cause higher Na^+^ accumulation and locally depolarize perisynaptic astrocyte processes due to increased ${\left[{\text{K}}^{+}\right]}_{\text{o}}$, caused by postsynaptic currents.

The proposed model is primarily based on experimental data for cardiac NCX1 isoform (Matsuoka et al., 1996; Ginsburg et al., 2013), because there is no detailed experimental data on kinetics and modulation available for astrocytic NCX. Astrocytes do express NCX1 (Minelli et al., 2007), but other NCX types are also present, and even for the same isoform, kinetic properties may be tissue dependent. Nevertheless, the predictions of the model look compelling enough in the context of astrocytic Ca^2+^ signaling to justify a dedicated experimental testing for the predicted nonlinear interaction between NCX and Ca^2+^ under high-Na^+^ and low-Na^+^ conditions.

## Data availability statement

Model implementation and parameter values used in the simulations are supplemented as a Jupyter (Kluyver et al., 2016) notebook available online at https://zenodo.org/record/1218115.

## Author contributions

AB: model formulation, preparing the figures, writing the paper; AV: model simulations, preparing the figures; DV: model simulations, preparing the Supplementary Materials; DP: model simulations, phase-plane analysis, writing the paper.

### Conflict of interest statement

The authors declare that the research was conducted in the absence of any commercial or financial relationships that could be construed as a potential conflict of interest.
