# Supplementary material for: Sodium–Calcium Exchanger Can Account for Regenerative Ca2+ Entry in Thin Astrocyte Processes
Source: Front Cell Neurosci. 2018 Aug 14;12:250. doi: 10.3389/fncel.2018.00250 (PMC6102320; doi:10.3389/fncel.2018.00250)
Supplement: Supplementary file 1 [file Presentation_1.PDF]

Supplementary material for “Sodium-calcium exchanger can account for regenerative calcium entry in thin astrocyte processes”

Alexey Brazhe, Andrey Verisokin, Darya Verveyko, Dmitry Postnov

June 10, 2018

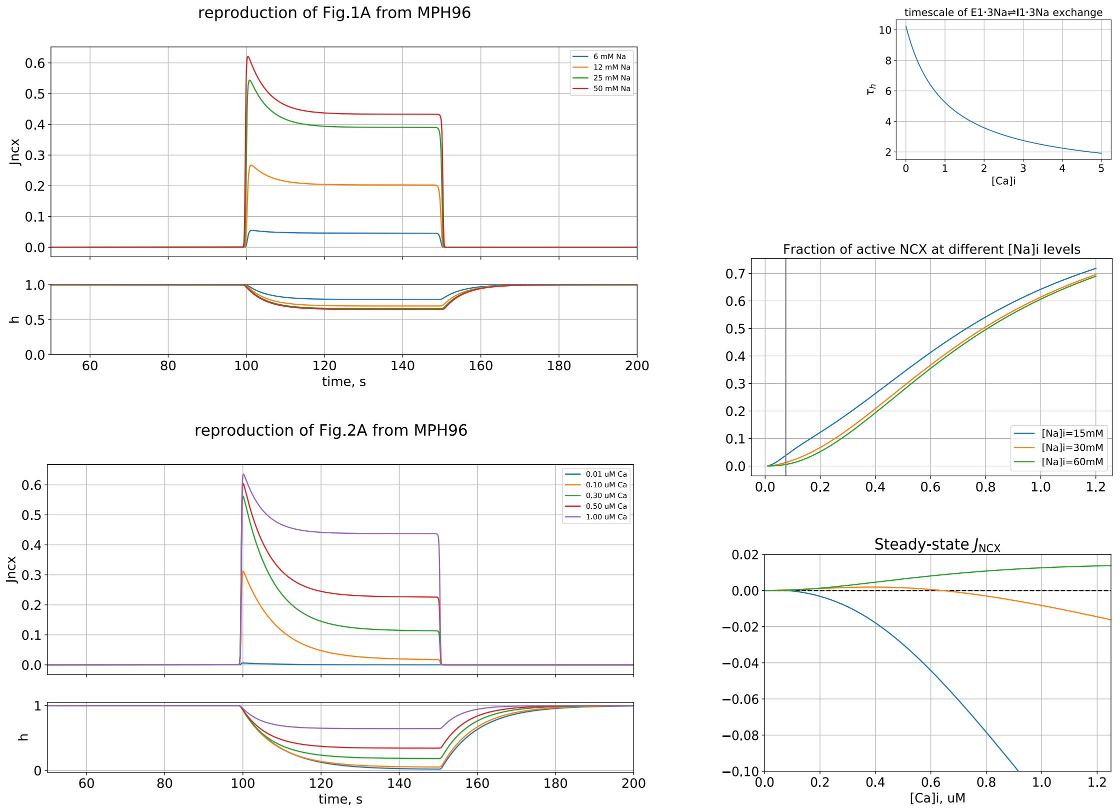

Figure 1: Detals of the model. Shown are reproductions of figures 1A and 2A from Matsuoka et al 1996, as well as dependence of the τ_h timescale on $[Ca^{2+}]_i$, fraction of active NCX molecules and relative flux through NCX at different $[Ca^{2+}]_i$ and $[Na^+]_i$levels.

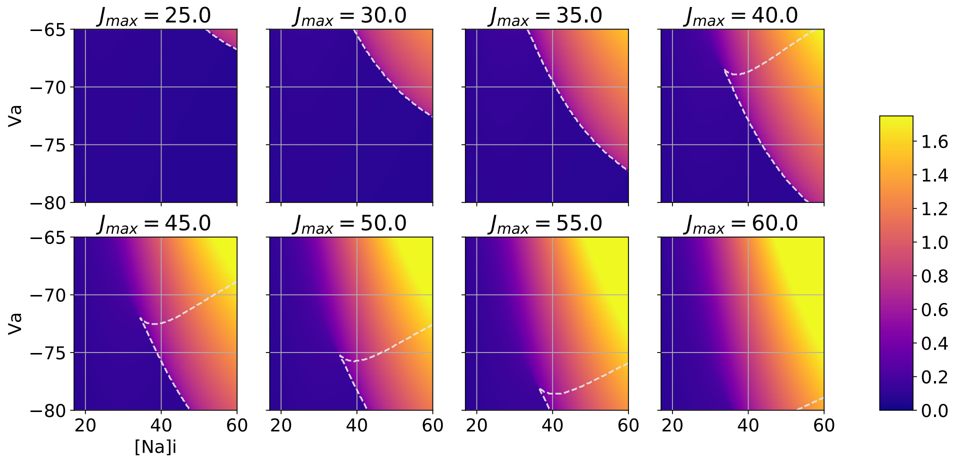

Figure 2: Maps of regions with bistable behavior (bounded by dashed white lines) and largest $[Ca^{2+}]_i$ in a stable equilibrium state.

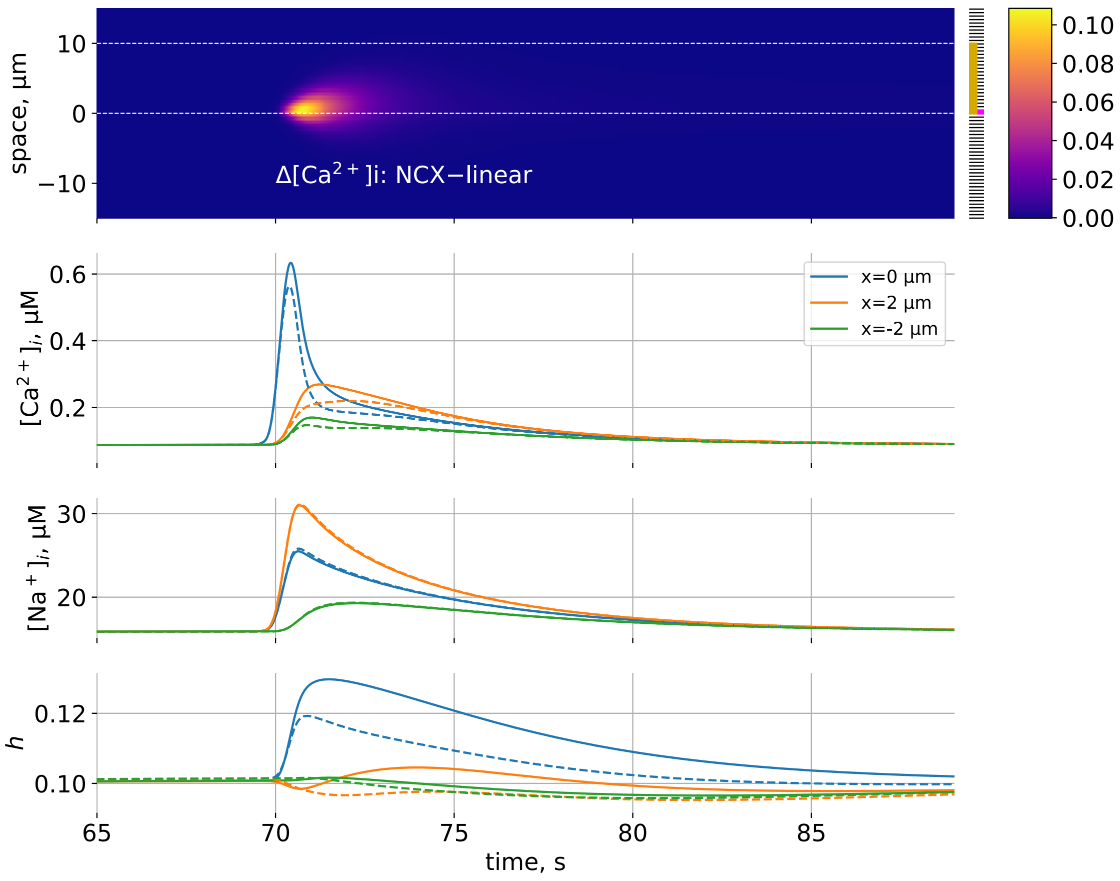

Figure 3: **Image:** *difference* between $[Ca^{2+}]_i$ reponse of proposed model to simultaneous presentation of Ca^{2+} and Na^+ stimuli and linear sum of responses to separate Ca^{2+} and Na^+ stimuli. Space discretization is shown to the right. Segments with Na^+ pulse are highlighted in yellow, segment with Ca^{2+} is highlighet in red. **Kinetics:** Model variables at three different locations; solid lines: response to simultaneous Ca^{2+} and Na^+ pulses; dashed lines: linear superposition of responses to independent pulses of Ca^{2+} and Na^+.
